# Supplementary figures and images for: Marmosets mutually compensate for differences in rhythms when coordinating vigilance
Source: PLoS Comput Biol. 2024 May 15;20(5):e1012104. doi: 10.1371/journal.pcbi.1012104 (PMC11132515; doi:10.1371/journal.pcbi.1012104)

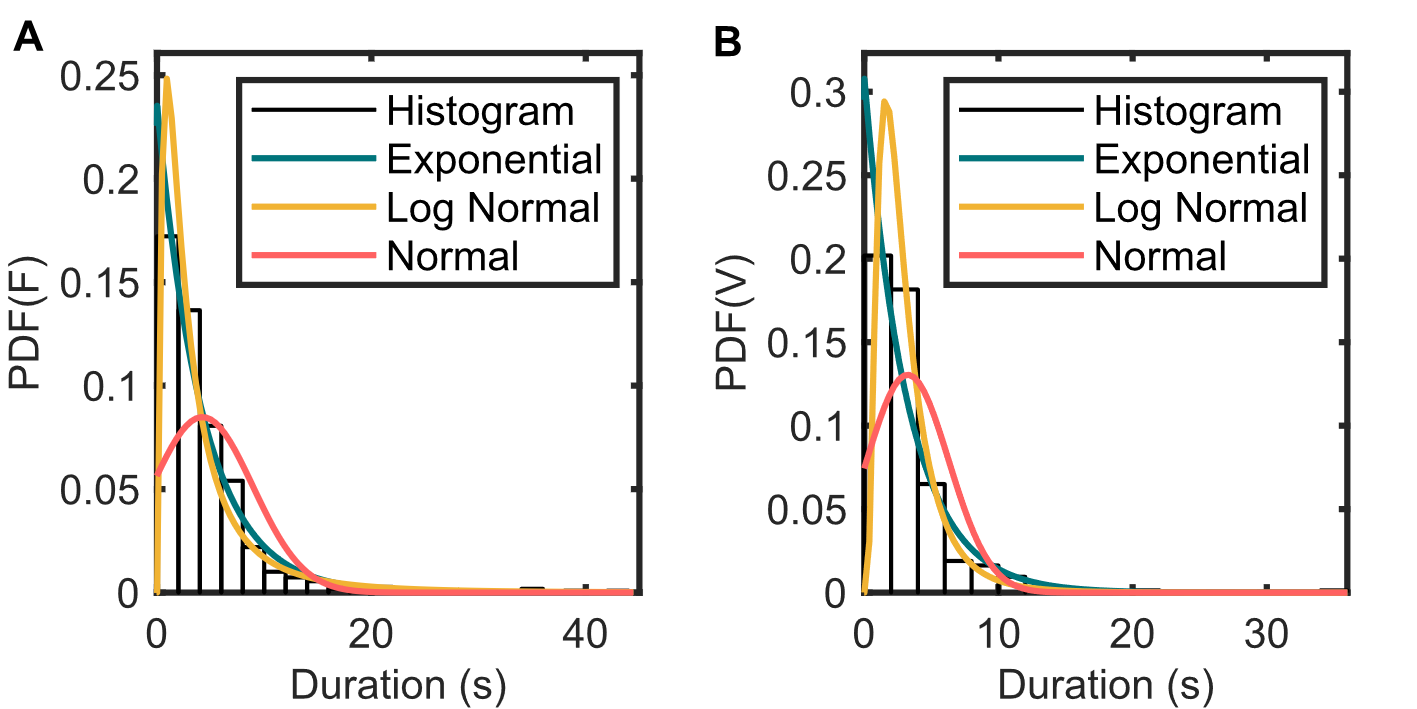

Supplement: S1 Fig — Plots depict histograms of the probability density functions (PDF) of feeding (A) and vigilance (B) durations across n = 14 individuals when they were alone, and negative exponential, log-normal and normal distributions fit to the histograms. (TIF) [file pcbi.1012104.s004.tif]

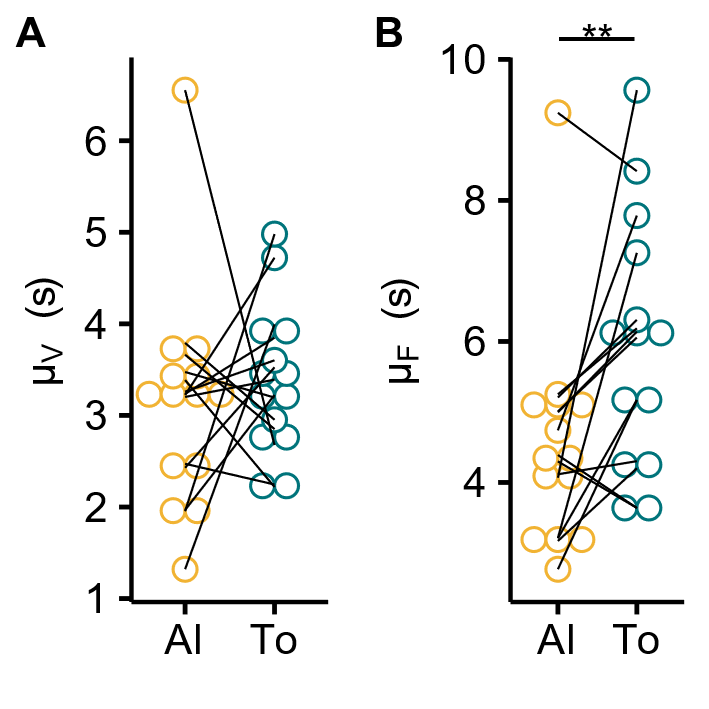

Supplement: S2 Fig — Plots compare the fit parameters of vigilance (A) and feeding durations (B) in the alone (Al) and together (To) conditions. Each point is an individual (n = 14 individuals). Individuals belonging to the same group are connected by lines. **p<0.01, two-sided Wilcoxon signed-rank test. (TIF) [file pcbi.1012104.s005.tif]

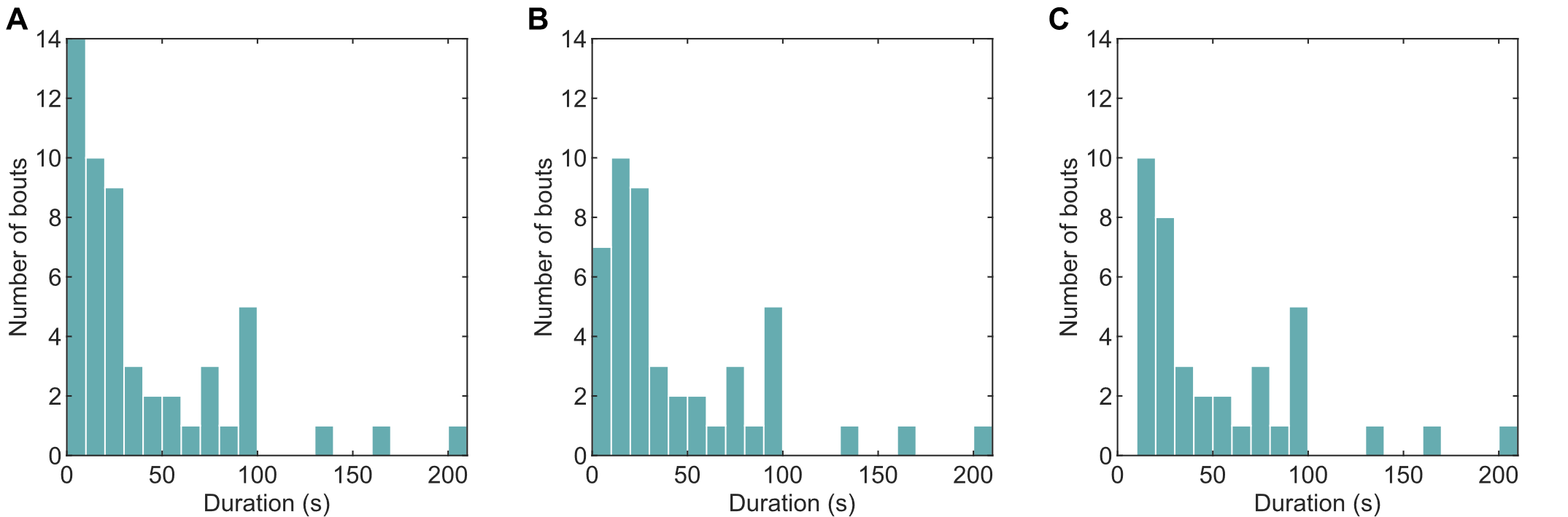

Supplement: S3 Fig — Histograms for all bouts in the data (A), bouts for which the Kuramoto model could be fit (B), and analyzed bouts (C). (TIF) [file pcbi.1012104.s006.tif]

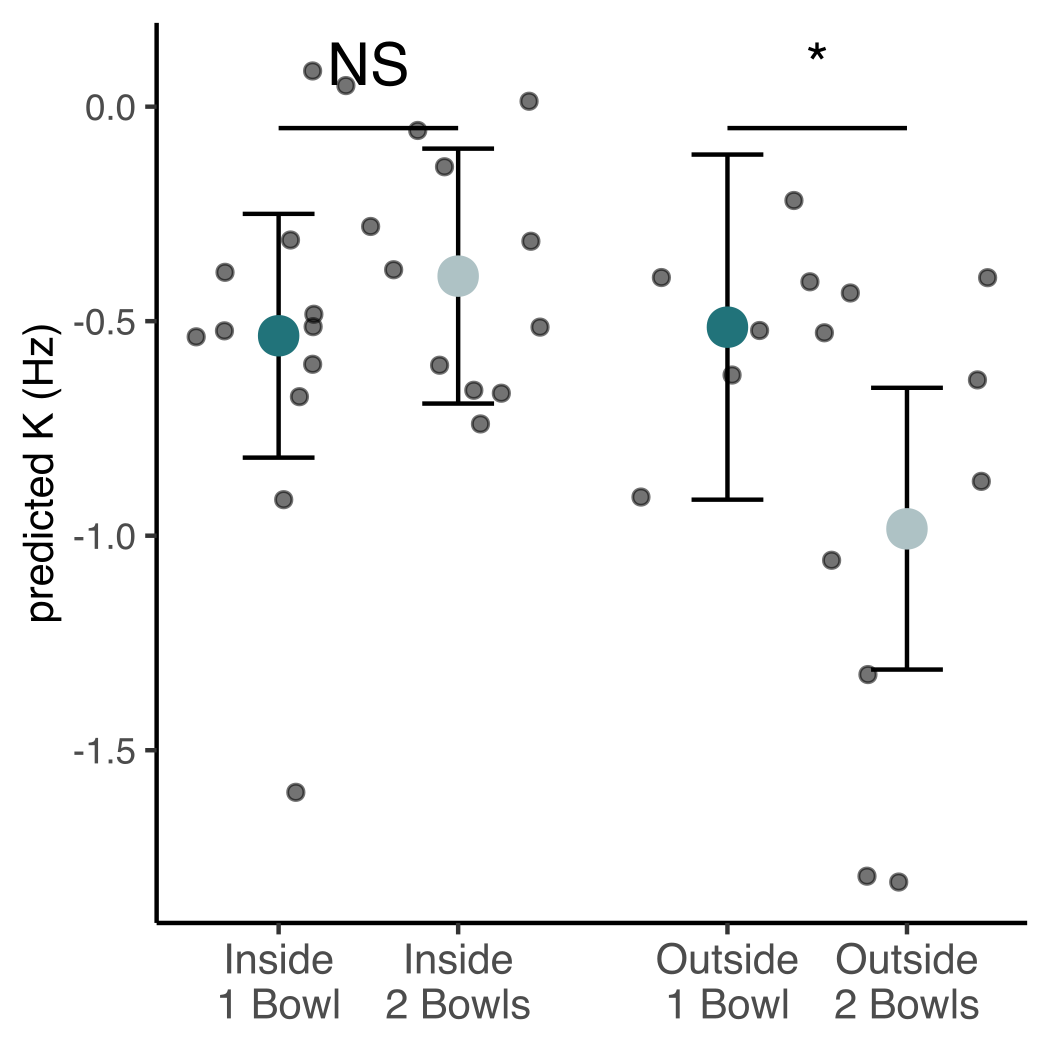

Supplement: S4 Fig — Each point is a behavioral bout (n = 38 bouts). Dots with error bars depict the estimated means and 95% confidence intervals. *p<0.05, NS = p>0.05, post-hoc comparisons of estimated means (Welch’s t-test). (TIFF) [file pcbi.1012104.s007.tiff]

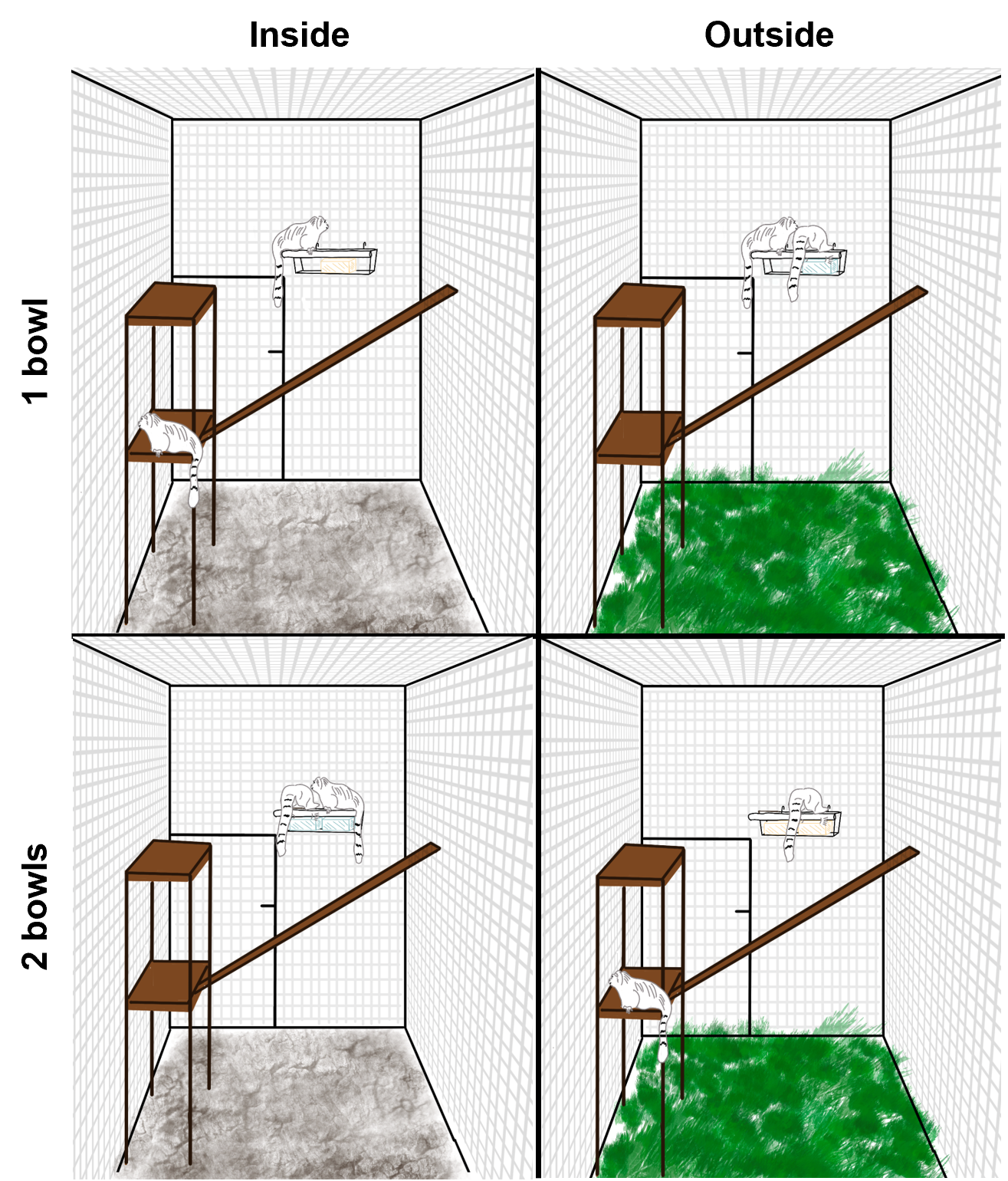

Supplement: S5 Fig — This schematic shows the view from within home enclosures. Individuals experienced either of these conditions (inside 1 bowl, inside 2 bowl, outside 1 bowl, outside 2 bowls) in a randomized order (see S3 Table) in their respective home enclosures. Animal were fed from either 1 or 2 feeding bowls located inside a feeding basket on the front of enclosures. During the whole experimental duration, animals were able to move freely within the respective location. Cameras were place both outside and inside of home enclosures (not shown). Video coding was done frame by frame according to definitions from S2 Table and specifically included all looking behavior over arms reach and not at a conspecific in any location of the enclosure as well as feeding behavior. For data analysis the datasets were restricted to either the “together” condition (blue bowls) where animals were situated together on the feeding basket or “alone” condition (yellow bowls) where animals were situated alone on the feeding basket. Note that even though “together” and “alone” conditions are only shown for two out of four possible conditions animals’ data was present for all four conditions. Note that the marmosets are depicted disproportionally large relative to the size of the overall enclosures. (TIF) [file pcbi.1012104.s008.tif]
