# Supplementary material for: Marmosets mutually compensate for differences in rhythms when coordinating vigilance
Source: PLoS Comput Biol. 2024 May 15;20(5):e1012104. doi: 10.1371/journal.pcbi.1012104 (PMC11132515; doi:10.1371/journal.pcbi.1012104)
Supplement: Attachment — Submitted filename: Vigilance_PLOSCB_responses1.pdf [file pcbi.1012104.s009.pdf]

University of Zurich[UZH]

Institute of Evolutionary Anthropology

University of Zurich
Irchel Campus
Winterthurerstrasse 190
8057 Zurich
Switzerland
www.aim.uzh.ch/de.html

Nikhil Phaniraj
Phone +41 44 635 54 27
nikhil.phaniraj@uzh.ch

Zürich
24.01.2024

The Editor
PLOS Computational Biology
Nine Hills Road
Cambridge CB2 1GE, United Kingdom

Responses to reviewers' comments for "Marmosets mutually compensate for differences in rhythms when coordinating vigilance."

Dear Dr. Zhang and Dr. Simon,
We would like to thank you for giving us the opportunity to resubmit our manuscript titled "Marmosets mutually compensate for differences in rhythms when coordinating vigilance" to *PLOS Computational Biology.* We sincerely appreciate the time and effort you and the reviewers have dedicated to providing valuable feedback on our manuscript. We are grateful to the reviewers for their insightful comments on our paper, which have helped us revise it significantly. Firstly, we have reformatted the structure of the manuscript according to *PLOS Computational Biology's* guidelines. This included moving the methods section to be placed after the discussion section. More importantly, we have been able to incorporate changes to reflect all the suggestions provided by the reviewers, and we genuinely believe that the quality of the manuscript has substantially improved upon these revisions. Here is a point-by-point response to the reviewers' comments and concerns, and the related changes to the manuscript (highlighted).

Reviewer #1:
This paper presents models and data to determine how animals coordinate their vigilance. The paper shows evidence that marmosets in pairs coordinated their vigilance and that this was achieved through mutual adaptation as the rhythm of each individual differed when they were alone.

The paper makes both empirical and theoretical contributions, which is quite rare and highly relevant to a computational journal. I can see the value of adapting the classic Kuramoto model to non-periodic cases. However, I am not able to determine whether this was done correctly as the mathematics eluded me. Most of my comments below are thus directed at the biology. Many comments probably underscore my lack of knowledge about this type of modelling. At the same time, if the paper is to reach a behavioural ecologist like myself, some changes are needed. Nevertheless, I think it is fascinating to use coupled oscillators to investigate synchrony in vigilance. This is much better than the simple approach used in the past, which looks at group level patterns but eschews individual adjustments.

Response: We sincerely thank reviewer 1 for taking the time and effort to assess our paper in detail. We truly appreciate their valuable feedback and are glad that they recognize our efforts to combine an empirical and theoretical contribution to the field of animal vigilance. We hope that the changes we introduced to the text make it more clear to a reader with a minimal mathematical/modelling background.

Line 86 : Should this not be anti-phase synchronization?
Response: Thank you for this observation, yes anti-phase synchronization makes more sense here and has been changed accordingly see line 105.

> Intriguingly, vigilance can also be coordinated in anti-phase synchronization (i.e., animals maximizing the feeding time when any group member is vigilant [52]), leading to turn-taking-like patterns [53–

55].

Line 88 : The evidence for coordination is actually quite good for sentinel-like systems. Evidence for cooperation is less common in non-sentinel systems when all individuals are actively foraging. It typically involves small groups like families in cranes or pairs in coral fish. Perhaps expand on this a little.
Response: We fully agree with this comment and have added a sentence on coordinated vigilance outside of sentinel systems, see lines 105–108.

> Intriguingly, vigilance can also be coordinated in anti-phase synchronization (i.e., animals maximizing the feeding time when any group member is vigilant [52]), leading to turn-taking-like patterns [53–55]. Such evidence for anti-phase synchrony is most prevalent in groups with sentinel systems and otherwise typically restricted to (mating) pairs (coral reef fish: [56,57]; birds [58,59]).

Line 106: Are there alternatives to the Kuramoto model? Please justify this choice a little more. Also, explain what it means when oscillators are coupled? Perhaps give some examples when this sort of coupling occurs. I have a hard time imagining how oscillators can be coupled for non-living oscillators and evolve to a steady state.
Response: The first mathematical model for a system of interacting oscillators was proposed by Arthur T. Winfree. However, the general Winfree model is not exactly solvable.
Yoshiki Kuramoto was inspired by Winfree's formulation and came up with a mathematical formulation that is solvable. Even though proposed in 1975, Kuramoto's model is till date the most successful attempt at characterizing coupled oscillator systems. All further work and modeling of coupled oscillator systems after 1975 have been slight modifications of the Kuramoto model to suit the nature of the oscillator under study. Some popular modifications include the integrate-and-fire model for pulse coupled oscillators (when the coupling/interaction between the oscillators occurs only momentarily during a cycle such as firefly flashes), and the second order Kuramoto model for oscillators with inertia (such as power grids). As our system does not fit these special cases, we started with the classic Kuramoto model. Our modification of the classic Kuramoto model to encompass non-periodic oscillators is a continued effort to extend the applicability of this solvable system of equations for a wider range of synchronization phenomena.

Two oscillators are said to be 'coupled' if they interact in such a way that they influence the behavior of each other. For example, metronomes that can interact with each other through a common, freely movable platform evolve to a steady state of in-phase synchrony (see: https://youtu.be/T58lGKREubo?si=GTOk4CqWipngbTyC)

We have now mentioned the popularly used alternatives (i.e., modifications) to the Kuramoto model and justified why we used the classic model in lines 129-135.

> The Kuramoto model is one way to model the development of synchrony and its temporal variations. Other popularly used models include the integrate-and-fire model for pulse coupled oscillators (when the oscillators interact only momentarily during a cycle such as firefly flashes) [70] and the second order Kuramoto model for oscillators with inertia (such as power grids) [71]. As our system does not fit these special cases, we started modeling our system using the classic Kuramoto model. However, the classic model assumes that the individual oscillators are inherently periodic. We thus additionally developed a non-periodic version of the Kuramoto model, as many biological systems are not expected to be inherently periodic [73,74].

We also define the word 'coupling' in the introduction lines 60-64.

> There is extensive evidence from invertebrates that synchronized patterns can emerge as epiphenomena of animals following very simple rules: fireflies, for example, simply flash sooner than usual whenever a neighbor flashes and the resulting synchrony emerges from these small changes in local interactions [11–13]. In such cases, where individuals are not synchronized to begin with; individuals need to interact in such a way that they influence the behavior of each other, i.e., 'couple', to evolve to a synchronized state.

Line 120 : Many models predict regular vigilance, meaning that the duration of vigilance bouts is centred on a particular value rather than taking on a wide range of values. This would lead to quite periodic oscillations I would think. What is the evidence that marmoset vigilance is not regular?

Response: True, regular vigilance would lead to periodic oscillations. However, for marmosets, we find that feeding (F) and vigilance (V) intervals follow an exponential distribution (see figure below).

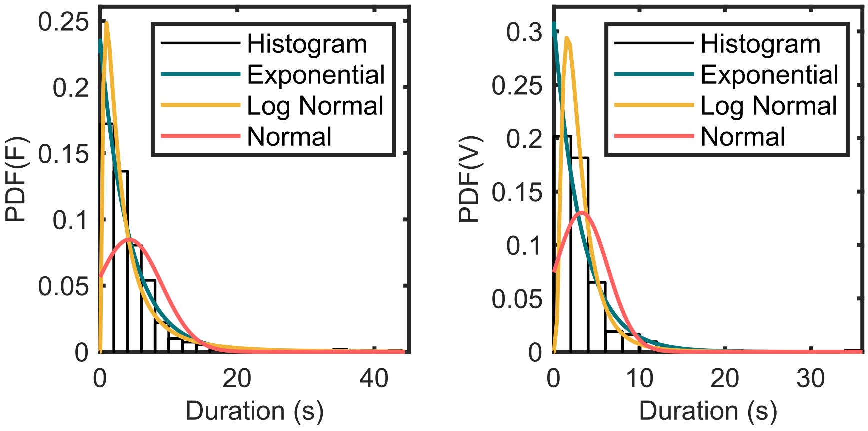

As we had only provided the vigilance/feeding distributions of one example pair in Fig. 3A, we now have included the above plots, which show the distributions of all individuals, as figure S1.

Line 158 : This could be a good place to provide details about the feeding apparatus so that the reader can understand how vigilance and feeding are mutually exclusive.

Response: Vigilance and feeding are mutually exclusive because the mash the animals were eating in experimental sessions is of a consistency that only allows licking up. Whenever marmosets are feeding their head has to be fully inside the feeding bowl which was of a size that the rim of the bowl would cover their eyes, making it impossible for them to scan the surroundings at the same time. It is debatable who much the animals are consciously aware of this. Answering this question would require further experiments with varying degree of head coverage to get at the more detailed mechanisms. We now include the crucial additional information about the feeding bowls in lines 484-487:

> Moreover, the food (mash) was only accessible via licking from the bottom feeding bowls. The rims of the bowls were higher than the head of the marmosets thus leading to full coverage of the eyes of the animals while feeding, making feeding and vigilance behavior mutually exclusive. For an overview of the experimental setup see Figure S5.

Line 160 : I doubt that potential predators are present near the enclosures but I suppose marmosets are not totally aware of this.

Response: We do not have systematic data available to quantify how many times raptors and cats are visible per hour from the outside enclosures as well as from inside. The animals have access to outdoor enclosures at appropriate weather conditions (see methods line XX) where the enclosures are not visually blocked from their surroundings. In addition, our facility has glass roofs that make birds of prey visible for many of the groups even from the inside, in summer these roofs are often covered by blinds (due to otherwise excessive heating of the building) but otherwise the sky is visible. The primate station is situated in a park like area where a large forest is in close range (ca. 300 m away). Switzerland has a substantial population of red kites (2800–3500 pairs (2013–16); for 2018 the breeding population grew by 59 % relative to the mean population value over the whole data collection period. Source: https://www.vogelwarte.ch/en/birds/birds-of-switzerland/red-kite) and kestrels are also frequent (5000–7000 pairs (2013–16), breeding bird relative population growth for 2018 was 37 %, source: https://www.vogelwarte.ch/en/birds/birds-of-

switzerland/common-kestrel). We observe cats move by the outside enclosures regularly (= at least twice a week) in summer (the park like area where the station is situated in is also surrounded by a neighborhood with many single-family houses). Additionally, there are drones that are flown within the park area especially during spring and summer. Foxes are also part of the park fauna but are seen less frequently by the monkeys since during the dawn and dusk hours monkeys are mostly spending time in the inside enclosures. While of course birds of prey are especially salient predator stimuli, the captive marmosets studied here are even giving aerial alarm calls to doves, pigeons and crows which are very frequent in the park (during nice weather conditions certainly 1–2 birds seen in 15 min).
We now include a short mention of types of prey that can be seen from outside enclosures see line 478–479 now reads:

> where potential predators (such as cats, birds of prey or more rarely foxes) were visible more frequently, leading to a “higher risk” situation.

We additionally mention these observations in the introduction (lines 123–125):

> Even in captive settings marmosets maintain high levels of vigilance, as for instance when responding to unfamiliar humans with antipredator behavior and emitting warning calls upon spotting birds of prey (personal observations by RKB & JMB).

Line 166 : Did you watch the videos frame by frame to determine the beginning and ending of behavioural bouts and if so what was the frame rate?
Response: Yes, video coding was done frame by frame especially since the target behaviour included all looking behaviour over arms reach (see also Ethogram Table S2). The frame rate was 25 fps (this was already mentioned in line 577) but we now describe the coding process more accurately including the frame rate of the video already when describing the video coding. This section now reads (lines 500–502):

> We coded three behaviours frame by frame (videos were recorded with 25 fps) namely: vigilance, feeding and out of sight as well as the locations of the animals (inside/outside or on basket) according to the detailed definitions in Table S2.

Line 196 : I was lost here with this sort of transformation. It was not totally clear to me why this was needed.
Response: Because we coded the videos frame by frame, the transitions between vigilance and feeding behaviors in the data are instantaneous. See figure 5A and 5B for example, the resultant time series plots look like step functions. This is not ideal to fit the Kuramoto model which considers gradual transitions. Biologically, we are also aware that marmosets are unable to perform such quick transitions. The fastest head turns in marmosets are known to happen at about 1000 degrees per second. We used this value as an upper limit to transform the time series such that it shows smoother, gradual transitions between vigilance and feeding behaviors. We have explained this in lines 521-525.

> Because marmosets are known to show head movements of angular velocities up to 1000 degrees per second [97], which equates to 2.78 complete head oscillations per second, we lowpass filtered the time series using a filter of 2.78 Hz (using MATLAB’s ‘lowpass’ function from the ‘signal processing toolbox’). This smoothened the time series by modeling the transitions between the feeding and vigilance states to be gradual.

Line 199 : I was not sure what phase angles mean practically.
Response: Obtaining phase angles using the Hilbert transform helps mapping any periodic behavior onto a circle, in this case, mapping the head oscillations from the top-most point (‘vigilant’) to the bottom-most point (‘feeding’), back to the top-most point onto a circle.

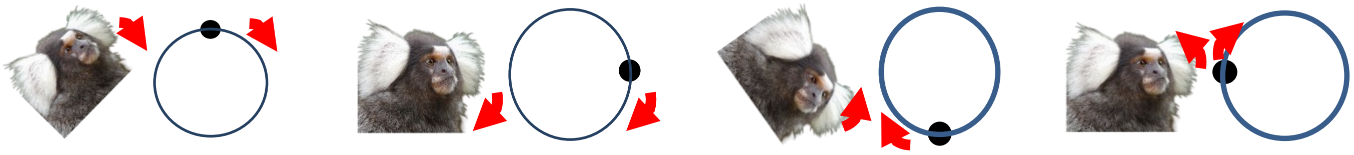

Now, every point on the circle, and consequently every head position, can be represented by a unique value of the angle subtended by the line joining the centre of the circle and that point, on the horizontal, which is the phase. So, during one complete head oscillation from the top-most to the bottom-most, back to the top-most point, the marmoset goes through phase values: π/2 -> 0 -> -π/2 -> π -> π/2 in radians. We have now explained this briefly in lines 525-529.

> A Hilbert transform was applied to the filtered time series (using MATLAB's 'hilbert' function from the 'signal processing toolbox'), and a time series of phase angles was obtained. This helped map marmoset head oscillations onto a circle wherein instantaneous head positions could be represented by unique phase angles ranging from -π to π radians.

Line 203 : As mentioned earlier, regular vigilance is also possible at least in other species. The Poisson model assumes that the rate of interruptions is constant, thus yielding a negative exponential distribution of duration lengths. Have you tried a normal distribution, which would fit with the regular model, or a log-normal, which might be appropriate under certain circumstances? At least make the modelling choice more transparent.

Response: Yes, the normal distribution provides a poorer fit. The log-normal distribution fit peaks very early (at about 0.9s for feeding durations and 1.9s for vigilance durations when some feeding/vigilance durations are up to 30s long) and then traces a curve very similar to the negative exponential distribution. Therefore, it did not make sense to replace a 1-parameter exponential distribution with a 2-parameter log-normal distribution when the former provided equally good, if not superior fit and the original distributions were monotonically decreasing.

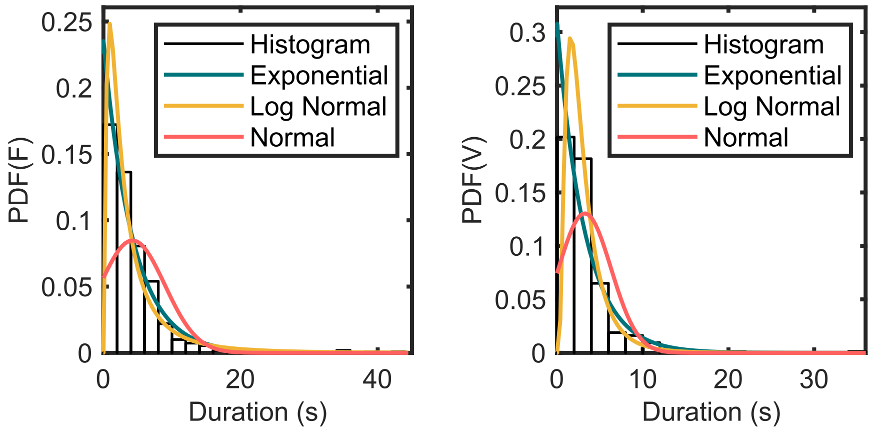

We have now provided a brief history of fitting functions for vigilance distributions and justified the fitting of the exponential function in lines 533-544.

> Previously, researchers have fit various probability distributions to infer the patterns underlying a species' vigilance behavior. The Normal, Log-Normal and Exponential distributions turn out to be the best fitting distributions under different cases [15]. In species that show a regular, periodic vigilance, characterized by a distinct peak in the distribution for a particular duration of vigilance, the Normal distribution turns outs to be the best fitting function. In some species, the vigilance distributions are right-skewed and the log-normal distribution provides a superior fit. Researchers have hypothesized that this in would indicate that various factors underlying vigilance behavior of the species have a multiplicative effect. In a few cases, the vigilance distributions are monotonically decreasing and the negative exponential distribution turns out to be the best fit. This would suggest a Poisson-like process with an underlying rate of switching between behaviors such that each switch is independent from previous switches. The distribution of vigilance durations for marmosets followed a monotonically decreasing function and we therefore fit exponential functions (using MATLAB's 'fitdist' function from the 'statistics and machine learning toolbox') to the distributions of durations.

Line 206 : I was lost here again. What is a ratio of fit parameters? Please define the fit parameter and then how this ratio was calculated. Also, explain why this is needed and how to interpret their values.
Response: Because the negative exponential distribution is a 1-parameter distribution represented by $y = \frac{1}{\mu}e^{-\frac{x}{\mu}}$, the fit parameter μ is simply the mean (feeding/vigilance) duration. The ratio of fit parameters is the ratio of μs obtained by fitting exponential distributions to feeding/vigilance durations of the two individuals in a pair. The fit parameter represents the distribution by capturing the decay rate and therefore, a ratio closer to 1 would indicate that the two individuals have similar distributions whereas a ratio much smaller than 1 would indicate that the two individuals have dissimilar distributions.

We have now defined what we mean by the fit parameter and its ratio in lines 545-551.

> The toolbox fits the function $y = \frac{1}{\mu}e^{-\frac{x}{\mu}}$, where the fit parameter μ is simply the mean duration. We studied the fit parameter for the two behavioral durations for all individuals, both when they were alone and when they were together. Specifically, we compared the ratio of the fit parameters of the two individuals of a pair for vigilant and feeding durations. A ratio closer to 1 would indicate that the two individuals have similar distributions whereas a ratio away from 1 would indicate that the two individuals have dissimilar distributions.

Line 222 : Please explain the terminology for this sort of model. I doubt many readers will know the meaning of terms like phase and natural frequency.
Response: Thank you for pointing this out. We have now defined phase and natural frequency in lines 562-567.
The classic Kuramoto model [69,70] (henceforth 'Kuramoto model') for N oscillators is represented by the equation:

$$\frac{d\theta_i}{dt} = \omega_i + \frac{K}{N}\sum_{j=1}^{N} \sin\left(\theta_j - \theta_i\right), \qquad i = 1 \dots N \qquad (1)$$

Wherein the ith oscillator completes its cycles at a rate of its natural frequency ω_i (in cycles per second = Hertz) and its cycles can be mapped onto a circle and its instantaneous state represented by the phase θ_i. K is the coupling constant and t is time.

Line 244 : With so many data points coming from the same individuals, there is a need I think to control for auto-correlation, no? This looks like a time series to me with all the trappings of correlated data.
Response: Here, by regressing $\Phi(t) - \Phi(t-1)$ over $sin\big(\Phi(t-1)\big)$ and obtaining the slope, we tried estimating the relative phase change undergone by the two individuals as a function of the phase difference between them at the previous timepoint. The idea is to determine at every instant (or in this case, in every analyze d frame), how the change in the behavior (i.e, the head position) of the individuals relates to their relative behavior at the previous instance. In this context, it is similar to obtaining the slope from a phase response curve (see Greenfield et al. (2021). *Philosophical Transactions of the Royal Society B*, *376*(1835), 20200338) wherein the instantaneous phase of the same individual (many data points) is plotted against the instantaneous phase of the stimulus when investigating stimulus entrainment. Mathematically, multiple data points from the same individual and the same behavioral bout is required to fit the Kuramoto model to the data.

Line 400 : Judging from the data distribution for both vigilance and feeding duration in figure 3, it appears that the fit to the exponential distribution must be rather poor as the distributions have humps. Like mentioned earlier, have you tried the normal or log-normal distributions? An overview of the different types of distributions that can be fitted with vigilance data please see Biology 11, 1769.

Response: Thank you for suggesting the reference. There is indeed individual variability in the distributions. However, overall, the marmosets seem to be following an exponential distribution. The humps in the distributions are majorly seen when in the 'together' condition. This change in the shape of the distribution when the individuals are together is accounted for by the interaction term in the non-periodic Kuramoto model.

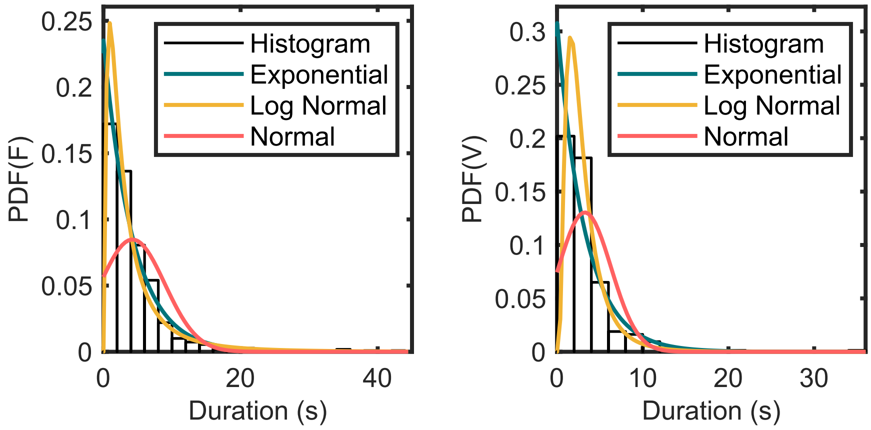

Line 404: Values closer to 1 suggest synchrony. This is not clear to me. This certainly means that the duration of vigilance bouts is now more similar when in pairs but does it really say anything about when those vigilance bouts occur for each individual? These bouts could still be totally asynchronized between pair members, no?

Response: Throughout the manuscript, we define synchronization between oscillators as the oscillators oscillating at the same rate, irrespective of the phase. In-phase and anti-phase synchrony are only two example cases of synchronization. Synchronization, in principle, can occur with any phase difference. We have now made this clearer in the introduction lines 49-57.

> Synchronicity not only includes patterns where individuals behave in the same way at the same time, as in the impressive synchronous light flashes of fireflies or coordinated movements of starling flocks [18]. Many phenomena show anti-phase synchronization in which individuals alternate behavior [19], as in vocal turn-taking in marmoset monkeys [20,21], meerkats [22], elephants [23] and plain-tailed wrens [24] or gestural exchanges of mother-infant dyads in chimpanzees and bonobos [25]. Whereas in-phase and anti-phase synchrony are the two extremes and frequently occur in nature, synchronization can, in principle, occur at any phase lag (e.g., [26]). Throughout this paper, we use 'synchronization' to refer to the phenomena of individuals showing a repetitive behavior at the same rate, with any phase difference between them.

Line 412 : In figure 3d, should you not compare the results controlling for pair id like in figure 3c? In the together condition, the data points are not independent.

Response: While figure 3b and 3c tells us that their distributions become similar when together, figure 3d tells us, whether at the level of the individual, they increase or decrease their rate of head oscillations. We compare, for every individual, the mean time period of head oscillations when they were alone vs when they were with another individual (their partner).

Line 415 : I am not entirely sure I understand the logic behind the argument that looking at mean time period of oscillations can tell us something about synchrony or coordination. Please elaborate.

Response: Here again, our definition of synchrony encompasses oscillators oscillating at the same rate irrespective of the phase difference between them. If the mean time period of oscillations of two oscillators are similar, it implies higher synchrony. Further, the increase in the mean time period of every individual when another individual is present points towards a phase delay mechanism (see Greenfield, M. D. (2005).

Advances in the Study of Behavior, *35*(05), 1-62.) indicative of the possible emergence of anti-phase synchrony. We only confirm this later, after fitting the Kuramoto model. We have discussed this result later in depth in the discussion section lines 337-350.

> Marmosets displayed changes in behaviors when they were together. The distributions of durations of being vigilant between the two individuals of a pair became more similar when together compared to when alone (figure 3B), a sign of synchrony. The feeding distributions also became more similar in the 'together' condition, even though this effect was not significant (figure 3C). Overall, the mean time period of oscillations (vigilance and feeding) of the two individuals of a pair were positively correlated when together, which was not the case when alone (figure 3E). This suggests that becoming similar to each other in vigilance durations along with a moderate level of similarity in feeding durations was sufficient to display overall synchrony. Moreover, marmosets spent longer in a behavioral state before changing their behavior when together, effectively slowing down head oscillations compared to when alone (figure 3D). Such slowing down of oscillations has been proposed to be a phase-delay mechanism of attaining anti-phase synchrony and has been described in case of alternating singing in several insect and frog species [1,19]. In katydid pairs for example, it has been proposed that a chirp from one individual delays the chirping of the other individual by a tiny bit (less than one cycle), thus inducing a phase-delay [80]. This applies symmetrically to both individuals and over time the individuals display anti-phase synchrony, with the frequency of chirping of each individual reduced.

Line 436 : Much of what is shown here was already established in the methods section. I think this section should be moved there.

Response: In this section, we propose a novel model that can explain the emergence of synchrony (both in-phase and anti-phase) in non-periodic oscillators. This model is general and can be applied to oscillators following ANY distribution. This development is a major result from a mathematical/computational biology perspective and hence we found it to be relevant to the results section. The model described in the methods section is a specific case of this very general model, fine-tuned to marmoset vigilance behavior. The equations in the methods section are not generalizable to other systems whereas the ones described in the results can be generalized.

Additionally, since in the revised version of the manuscript, the methods appear after the discussion, we prefer to keep this section where it is.

Line 437 : Could you not simply run a standard time series analysis to determine if oscillations were periodic when the individuals were alone? If this is the case, we would not need the non-periodic Kuramoto model.

Response: Yes, this can be done. However, because the distribution of feeding and vigilance durations follows an exponential-like distribution, we thought this was not needed. If the distributions were normal distributions with a very small variance, then the non-periodic Kuramoto model would not be needed.

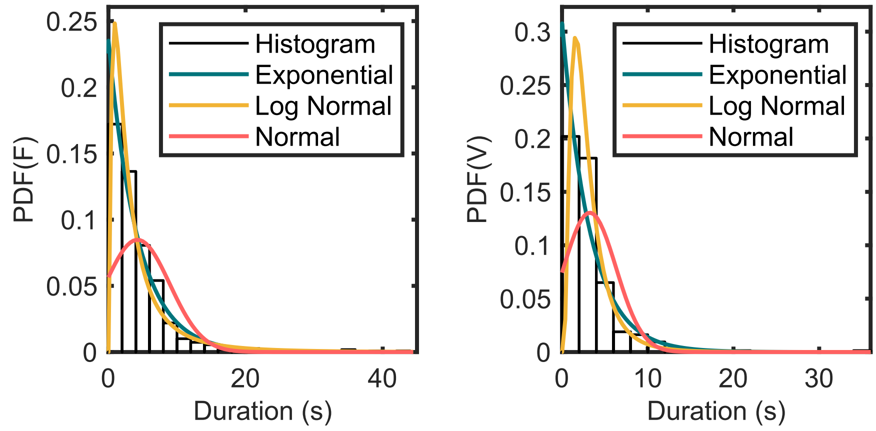

Figure 4 : Please explain which angles are compatible with synchronization or coordination.

Response: 0 degrees is compatible with in-phase synchronization whereas 180 degrees is compatible with anti-phase synchronization (coordination). We have now made this clear in the figure 3 (formerly figure 4) legend.

> A phase difference of 0 corresponds to in-phase synchrony, and that of 180° (π radians) corresponds to anti-phase synchrony.

Line 521 : I am not sure how we can conclude that the oscillations were non-periodic. In the results, I saw that the K constant was not the same for the periodic and non-periodic models. I cannot see how this can be used to assess model fit. Is there a way to show with a p-value which model best fitted the data?

Response: The oscillations were non-periodic because the distributions of feeding/vigilance durations were spread across a wide range of values and showed an exponential-like nature (as opposed to being spread across a narrow range of values and being Gaussian in nature). We did not use the K constant to assess model fits. We knew, even before fitting the classic Kuramoto model that our system does not obey the assumption of the model that the oscillators need to be periodic. Naturally, we expected the non-periodic Kuramoto model that does not have such assumptions to be a better model for our system. We previously did not compare model fits because both models were fit differently. The non-periodic model was not 'fit' in the traditional sense. Instead, several behavioral bouts were simulated using the non-periodic model equations and the one that most resembled the empirical data was chosen to be the best model. However, now we realized that at their core, both models are trying to predict the relative phase between the two individuals at every instant. Therefore, we now devised a mean absolute error (MAE) metric for the two models. MAE is the mean of the absolute differences between the instantaneous relative phase observed, and that predicted by the model. As expected, the non-periodic Kuramoto model had significantly lower MAE values ($p<0.001$, signed rank = 739, Wilcoxon signed rank test). The bar plot below shows the mean +- s.d. MAE for the classic and the nonperiodic models.

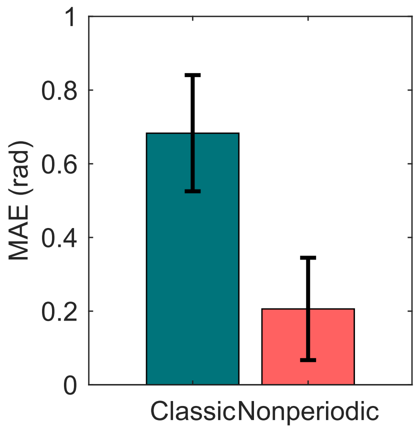

We have described the devised error in the methods section lines 697-703,

> *Comparing models*
>
> We compared the K values obtained by fitting the non-periodic Kuramoto model to the actual bouts to that of the values obtained by fitting the classic Kuramoto model to the actual and control bouts. Further, to assess model fits, we devised a Mean Average Error (MAE) metric which is the mean (averaged over a behavioral bout) of the absolute differences between the observed instantaneous relative phase $\phi(t)$ in radians and that predicted by the model. We compared MAEs of the classic and non-periodic Kuramoto models fit to the actual bouts.

the outcome in the results section lines 287-289,

> As expected, the non-periodic Kuramoto model provided a superior fit compared to the classic model with significantly lower Mean Average Error (MAE) values (figure 5D, N=39 behavioral bouts, signed rank = 739, $p<0.001$, Wilcoxon signed rank test).

and included the figure as a subplot in figure 4D.

Line 533 : As stated earlier, I am not fully convinced that the exponential is the best fit to the data. Also, it would be nice to discuss the rich literature on fitting functions to the distribution of vigilance duration. In many cases, the exponential was a poorer fit than other distributions. Exponential-like distributions are expected when individuals face stalking predators that can approach surreptitiously when foragers are not vigilant. Would this apply to marmosets? Just to be clear, it is the probability of changing state that is independent of the duration of the preceding state.

Response: Indeed, an exponential distribution would mean that the probability of changing the state is independent of the duration of the preceding state. We find that marmosets follow an exponential distribution and that would point to the possibility that marmosets have an underlying rate of changing state, but there is no memory effect (i.e., the vigilance bouts are not influenced by previous bouts).

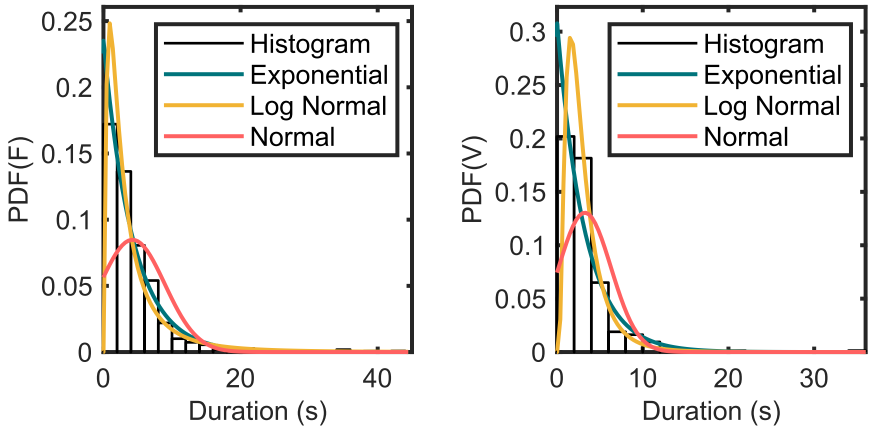

We have now discussed some of the literature on fitting functions for vigilance behaviors in lines 533-542.

> Previously, researchers have fit various probability distributions to infer the patterns underlying a species' vigilance behavior. The Normal, Log-Normal and Exponential distributions turn out to be the best fitting distributions under different cases [15]. In species that show a regular, periodic vigilance, characterized by a distinct peak in the distribution for a particular duration of vigilance, the Normal distribution turns outs to be the best fitting function. In some species, the vigilance distributions are right-skewed and the log-normal distribution provides a superior fit. Researchers have hypothesized that this in would indicate that various factors underlying vigilance behavior of the species have a multiplicative effect. In a few cases, the vigilance distributions are monotonically decreasing and the negative exponential distribution turns out to be the best fit. This would suggest a Poisson-like process with an underlying rate of switching between behaviors such that each switch is independent from previous switches.

Line 542: We would expect vigilance to go down when animals are in pairs compared to when they are alone and also to spend more time feeding. Therefore, I was not expecting the results that marmosets were spending more time in each behavioural state when together. This should be true only for feeding duration. From figure 3a, one individual was more vigilant when in pair than alone. Was it a common finding? It would be nice to see what happens to mean duration of vigilance and feeding when alone or in pairs to judge the group-size effect. If the individuals converge in vigilance time, we would expect that each one could only feed for 50% of the time if coordinated, perhaps a value lower than when some marmosets were alone. Did this happen in these pairs and if so it would indicate a cost of coordination.

Response: We agree. In general, as the group size increases, we would expect the time spent by each individual being vigilant to go down. However, we think that going from n=1 to n=2 individuals is special. When alone, there is no way for the individual to feed and be vigilant at the same time. Therefore, the individual is vulnerable while it is feeding. When n=2, the individuals will benefit if they coordinate their behaviors such that when one individual is feeding, the other is vigilant, and vice-versa. This ensures that there is at least one individual who is vigilant at all times (and such a scenario is not possible when alone). This will not however lead to decrease in vigilance as they still have to be vigilant for the duration that the other individual is feeding. i.e., if the feeding bouts get longer, the vigilance durations also increase, which is what we see. When n increases to 3, only one individual would need to be vigilant when the other two are feeding. Here, there is a possibility to double the time one individual is feeding while keeping vigilance similar or, decrease vigilance to 50% while keeping the feeding duration same. Similarly, when n further increases (>=4), we would expect vigilance to further go down. A stepwise increase of group size is an obvious next step for our line of research.

On average, we do find that individuals were more vigilant when in pair than alone. But this difference is not significant (see Fig S2A). The feeding time significantly increased (Fig S2B).

Line 544: A slowing down of oscillations when in pairs does not require monitoring the state of the

companion. As per the group-size effect on vigilance and feeding, either a reduction in vigilance time and/or an increase in feeding time would be sufficient.
Response: We think that only a reduction in vigilance time and/or increase in feeding time would not be helpful, if, for example, the animals are showing these behaviors at the same time. If two individuals increase their feeding time but are feeding at the same time, they are both vulnerable. It would be helpful if the individuals coordinate their behaviors such that when one individual is feeding, the other individual is vigilant. However, coordinating such behaviors is hard when the individuals are not periodic, i.e., when it is hard to predict when one individual would finish feeding so that the other individual can start to feed. People have shown that such coordination can be attained gradually if the two individuals slow down their oscillations (i.e., add a phase delay) until they both reach a state of same oscillation frequency (synchronized state) but 180 degrees out of phase (anti-phase synchrony).

Line 555: Please provide references to the safety argument. It gives the impression that the authors are the first to have thought of this.
Response: We have provided a selection of references to support the argument (see lines 355–357).

> As to why marmosets may remain in a state for prolonged duration when together, an ethological explanation could be that a marmoset may feel 'safer' in presence of the other individual, hence prolonging the time it would feed [56,59,81,82].

Line 556: The ethological argument is a little sketchy. There is no reason a priori why individuals should synchronize or coordinate their vigilance. Maintaining independent vigilance would still provide benefits when individuals are in groups by reducing the time spent on vigilance and by increasing the time spent feeding. Some have argued that there is a benefit to synchronize vigilance (i.e. phase synchrony) if predators are more likely to target laggards (those less vigilant at the time of an attack). In this case, individuals want to avoid being the only one non-vigilant when others are vigilant. Therefore, they all tend to adopt the same state at the same time. This can lead to long periods when no one is vigilant. Coordination brings the most benefit at the group level by minimizing periods where no one is vigilant, but this comes at the cost of monitoring neighbours and ensuring that cheating does not occur.
Response: We agree that there is no a priori reason why individuals should/would need to synchronize or coordinate. However, because we found that marmosets indeed coordinate vigilance (see also [68]), we attempt to provide an ethologically relevant mechanistic (proximate) explanation of how such coordination might be achieved. Our data suggests that:

1. Marmosets coordinate vigilance.
2. Such coordination is only gradually achieved during a behavioral bout.
3. Marmosets increase both feeding time and vigilance time when together.

All of the above points to the phase delay mechanism of attaining anti-phase synchrony.

Line 572: But why is the coupling stronger when feeding from two bowls? The lack of space would suggest a weaker coupling when feeding from two bowls. Can they monitor one another more easily in on situation than the other?
Response: No, the marmosets can monitor one another equally well in both situations. Our argument here is that, if the feeding bowl was too small for two individuals to feed from one bowl at the same time (which they are not), it is possible that when one individual is feeding, the setup 'forces' the other individual to be vigilant due to the lack of feeding space, and the "strong-coupling" we see would simply be an artifact of the setup. However, we do not see stronger coupling in the case of 1 bowl.

We have made this clear both while explaining the setup in lines 479-487

> We provided one or two feeding bowls, this was necessary to account for the potential alternative explanation that turn-taking was externally imposed by space restriction, i.e. that the marmosets avoided to have the head inside the same feeding bowl simultaneously with their partner and therefore opportunistically engaged in vigilance while waiting. Critically, if the same turn taking

pattern also emerged in the two bowl condition, this alternative explanation can be excluded [68]. Moreover, the food (mash) was only accessible via licking from the bottom feeding bowls. The rims of the bowls were higher than the head of the marmosets thus leading to full coverage of the eyes of the animals while feeding, making feeding and vigilance behavior mutually exclusive. For an overview of the experimental setup see Figure S5.

And in discussion lines 375-378.

The experimental contrast between 1 vs 2 feeding bowls was included to control for the possibility that the animal would be forced to take turns because of a lack of space to feed together. This was clearly not the case, because turn-taking was also present, and indeed stronger, when feeding space was not restricted in the two bowl condition.

Line 584: How do we know that each individual changed its behaviour to coordinate with the other rather than one individual showing flexibility but not the other one? Did I miss the evidence for mutual adaptation rather than unilateral adaptation?

Response: In Fig 2D, we see that all individuals (except one) increased the time period of head oscillations (i.e., slowed down head oscillations) when together and, in the end their time periods are more similar to each other Fig 2E. If it was unilateral adaptation, we would not find such changes happening in all individuals.

Line 624: As mentioned earlier, I was not sure how it is possible to determine how one model, periodic or not, provides a better fit than the other.

Response: Thank you for pointing this out. We have now devised a mean absolute error metric to assess model fits. The non-periodic Kuramoto model gave a lower error, and hence a better fit. This plot is now included in figure 4D.

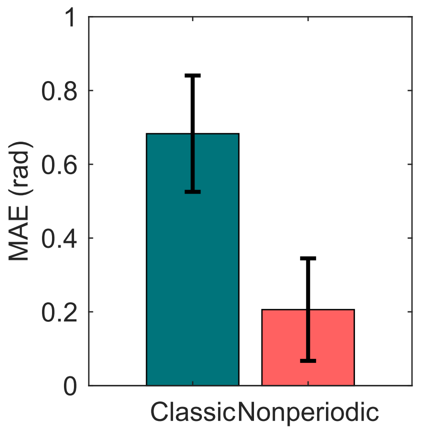

Reviewer #2:
The manuscript “Marmosets mutually compensate for differences in rhythms when coordinating vigilance” aims at modelling the vigilance behaviour of pairs of captive marmosets when feeding. The authors model the monkeys’ behaviour as (inherently aperiodic) coupled oscillators. The authors' main result is that marmosets take turns in their vigilance/feeding behaviours in a flexible way (i.e., variable coupling strength).

The study is very interesting, well-designed, and of potential interest to a wide audience. In addition, the authors extend the Kuramoto model to the case of non-periodic oscillators, which could be a very useful approach for many researchers. As I am not a modeller, however, I will refrain from commenting specifically on the modelling aspects of the manuscript.

Despite the positive points, I am uncertain about some methodological aspects, and—more in general—I think that the manuscript could be still improved in terms of clarity. In the following, I detail my concerns and questions to the authors.
Response: Thank you for going through our manuscript in great detail and suggesting changes to improve the intelligibility of our paper. We hope that, through our point-to-point responses to your comments and the related changes to the manuscript, you find all concerns to be resolved and the manuscript considerably improved and adequate for publication in *PLOS Computational Biology.*

Major points:
- The kind of behaviour the authors focus on is not clear to me. More specifically:
A) In the introduction, the authors broadly describe behaviours related to the sentinel system, but at line 120 they put the focus on “head oscillations” without having explained why. This should be better motivated and integrated with the previous part of the introduction.

B) While the focus here is on head oscillations, head movements were either not quantified or their quantification was not described, as the only coded relevant behaviour is “vigilance”. Could the authors explain better why they refer to head oscillations?

C) Did the authors collect any measure on the direction of the head (or eyes) movements?
Response to A, B, and C: The behavior we focus on is a change from looking behavior (what we term as *vigilance* defined in Table S2, because this is the term mostly used in the field) to feeding. The looking behavior we coded frame by frame is simply any looking over arms reach that is not directed at a conspecific (to exclude social vigilance, as this study focusses on anti-predator vigilance) or the substrate the animal is sitting on. For this definition of vigilance behavior (see Table S2) we follow a review by Allan & Hill (2018) [45]. They point out that a state of “vigilance” cannot be identified in animals as we cannot ask them if they are indeed trying to look out for predators. The only observable and thus identifiable behavior to researchers is thus looking behavior. Even if an animal does not consciously decide to be vigilant as long as its focus is over arms reach and not directly at another individual the likelihood of them being able to identify a predator is much higher than if their focus is on doing something else. The other behavior we additionally code (frame by frame as well) is feeding behavior which is defined as any moment in time the animal is having his/her head inside the food bowl.

What we then term “head oscillations” is simply the continuous change between the two states in question described in the paragraph above, i.e., vigilance and feeding. We use the term “oscillations” since it is a process of moving from one state to another and the body part that is moved during this process is the head. See also Figure in response to reviewer 1 for Line 199.

No, we did not collect any direct measure of the direction of the head and especially not eye movement due to two reasons: 1) Coding marmoset eye movements from videos with the quality we have recorded would be virtually impossible due to the small size of marmosets’ eyes and thus the difficulty to see the direction of

the eye. What was used as an indication of the looking direction of the animals while coding was the direction of the head and especially of the ear tufts which are very clearly visible and help to distinguish looking direction. 2) Our definition of vigilance as explained above includes basically any head direction that is not downwards and thus just by coding both vigilance and feeding behavior, we have all the necessary behaviors to model the coupled oscillators as we measure the "extremes" / "ends" of the oscillations. We then applied a Hilbert transform which allows to map any periodic behavior onto a circle (again see response to reviewer 1 for line 199)

-Lines 123-126: Please explain why you expect marmosets not to follow simple interaction rules during this behaviour.

Response: Such simple interaction rules are followed by insects like fireflies (for synchronizing flashes) and katydids (for synchronizing chirps) who follow a very stereotyped behavior (flashing/chirping). These insects have relatively simple visual and auditory processing systems. As marmosets have much complex sensorimotor systems and their vigilance behavior is not very stereotyped, we would expect them to couple more flexibly. We have now explained this in lines 176-182.

> Animals that show a stereotyped periodic behavior and eventually synchronize, such as fireflies flashing or katydids chirping seem to do so following simple, fixed interaction rules. If marmosets are following simple, fixed interaction rules to coordinate vigilance, we would expect the coupling strengths to be more-or-less uniform across feeding bouts. Moreover, we would expect the synchronization dynamics to be uniform. As marmoset vigilance behavior is known to be variable and not very stereotyped [67], we predict that there will be some variation in the coupling strengths across feeding bouts and that marmosets can synchronize flexibly.

-I assume the animals were born in captivity. Because of this, and since they are adult animals, I would expect them to know that no harm and no predators will come for them while they feed.

A) Can the authors (maybe in the discussion) speculate on whether they think the observed results would hold in wild animals, why yes or why not?

Response: It is true that all animals were born in captivity, and we go further into the details of how this captive setting might influence the vigilance behaviour of marmosets and include a paragraph on the implications of this setting in the discussion (see lines 406-416). See also answer to B) below.

> We acknowledge that this study has been conducted in captivity where vigilance is present [65,67,89–91] but chronic threat levels are much lower than in the wild. We hypothesize that under higher predation pressure, coordinated vigilance is even more likely to emerge since animals would experience much stronger trade-offs between the need to be vigilant and other activities. However, teasing apart the turn-taking coupling pattern in naturalistic conditions would not have been possible because wild marmosets hardly ever feed head-down without seeing anything at all (they feed on a wide-ranging diet, including insects, fruits, small mammals, but, importantly, also exudates [91]). This contrasts with the experimentally induced setting in this study, where the feeding individuals' views were entirely obstructed which allowed us to fully separate vigilance and feeding behavior. It would be highly desirable to complement the findings from our study with data from wild populations of common marmosets. Moreover, it is likely that vigilance coordination, both in captivity and in the wild, also occurs in other contexts, such as when infants are playing [93,94].

B) In line 160, the authors talk about a distinction between inside and outside as different risk zones based on the presence of "potential predators". Shouldn't the animal be habituated to both areas though, thus knowing no predators will come in either?

Response: It is true that animals are habituated to both the inside and outside home enclosures but importantly, animals in our colony are not routinely fed their morning mash in their outside enclosure. Thus, they were definitively less habituated to this feeding situation which we think contributed to their perception of the relative risk level. The captive setup of this study might first appear to represent a big disadvantage to studying vigilance behavior; after all, as you mention in your comment animals in captivity

might get habituated to the absence of predators and exhibit lower overall vigilance levels. Against this reasoning speak the numerous studies conducted on callitrichid vigilance in captivity and the observation in all of those, that vigilance behavior is part of the normal behavioral repertoire of captive callitrichids [65–67,89–91]. We often observe the animals in our colony to exhibit mobbing behavior towards birds of prey, drones or cats that can pass outdoor enclosures (all monkeys have access to outdoor enclosures). Additionally, we were in the very fortunate situation that data collection for this study happened not long after the facility moved to a new location with unfamiliar outdoor enclosures, i.e., a potentially dangerous environment in terms of captive conditions – so vigilance levels were expected to be sufficiently high, especially in the outside condition. Lastly during data collection for a pilot project, we could additionally quantify the amount of play displayed by two groups of marmosets between the inside and outside location. Play behavior in Golden Lion Tamarins has been shown to be displayed in less dangerous substrates such as the canopy, branches and floor compared to positions where they are more exposed to predators (large branches and vine tangles) as well as played more in the center of the group [93]. We found that both juveniles and adults virtually never played in the outside despite high levels of play inside ($p_{juvenile\ play/adult\ play}$ ~ location + condition + group: Results for juvenile play: $b_{location}$ (SE) = – 1.74 (0.45), 89 % CI [–2.47, –1.02], p– = 99.99 %. Results for adult play: $b_{location}$ (SE) = – 1.19 (0.72), 89 % CI [–2.37, –0.11], p– = 96.06 %; Fig. XX). This further strengthens our claims that the outdoor enclosures are perceived as less safe than the indoor enclosures or might possibly also represent rather the "periphery" of the territory of the marmosets in our colony.

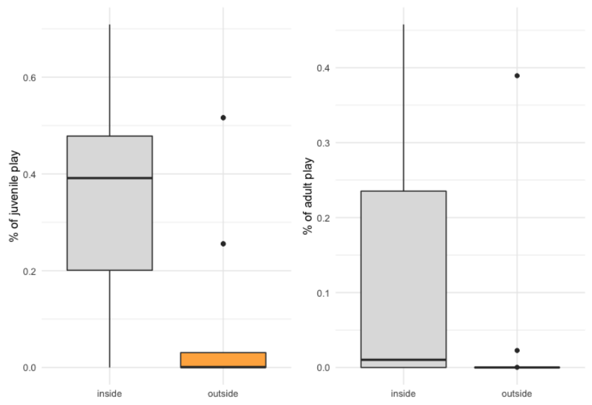

Comparison play behavior inside vs. outside pilot project. Differences between amount of play displayed in inside vs. outside enclosures by either juvenile (left hand side) or adult (right hand side) marmosets.

We now include a short elaboration about vigilance levels in our colony in the introduction (see lines 123-125).

> Even in captive settings marmosets maintain high levels of vigilance exhibited by responding to unfamiliar humans with antipredator behavior and emit warning calls upon spotting birds of prey (personal observations by RKB & JMB).

C) Related to the previous aspect, and because no direct measure of head/eyes movements was taken, I am wondering how we can be sure that the observed behaviour reflects vigilance rather than simpler taking turns to eat from the bowl. Could the authors elaborate on this?

Response: Even if we did not take any direct measurement of head/eye movements we did code for looking behavior which is specifically excluding looking at the partner and looking at the substrate. More importantly, the one-vs-two bowl feeding condition was explicitly included to exclude this alternative explanation. Animals would have the space and amount of food that allows them to eat all the time (until they finish the food provided), i.e., they can feed from one bowl simultaneously and specifically in the two bowl situation if their motivation to feed would be high they could easily just not be vigilant at all. But this is not what we observe: turn-taking occurs not only in the one bowl condition where it could be an artifact of reduced space but also in the two bowl condition where each individual has its own bowl. Furthermore, the

behaviors we coded are not just feeding and not feeding. The definition of vigilance excludes other behaviors such as walking around, inspecting enrichment in enclosures etc. Finally, as explained above (response to first major point A, B, C), there is no objective measure to quantify intentions that animals have of being "vigilant", in other words of if they want to look out for predators. What is operationalizable is only when animals are "looking" and thus would likely be able to spot predators (please see also answers below about how the behaviours on the basket relate to the situation during the experiment).
We now include some further explanations about why the 1 vs 2 bowl setup was included in our experimental design in lines 479–484:

> We provided one or two feeding bowls, this was necessary to account for the potential alternative explanation that turn-taking was externally imposed by space restriction, i.e. that the marmosets avoided to have the head inside the same feeding bowl simultaneously with their partner and therefore opportunistically engaged in vigilance while waiting. Critically, if the same turn taking pattern also emerged in the two bowl condition, this alternative explanation can be excluded [68].

- I am not sure I understand the control condition of the animal being alone in the feeding basket. Is it necessary for the companion animal to also be in the basket to exert vigilance on the surroundings? I would imagine that a nearby position would be equally effective. Maybe a picture of the real set-up, or a figure would be helpful here (see also my comments below).

Response: No, it is clearly not necessary for the pair mate to be on the feeding basket to be vigilant. While coding vigilance behavior from the videos animals could be situated anywhere in the enclosure for it to be included. This data then was analyzed in the "traditional" sense, where we most importantly showed, that when one animal was feeding, the other animal was more likely to be vigilant (and this data specifically included instances of vigilance anywhere in the enclosure) (see figure below copied from published paper [68]). This already hints at a probability of possible coordination of vigilance but is only indirect evidence.

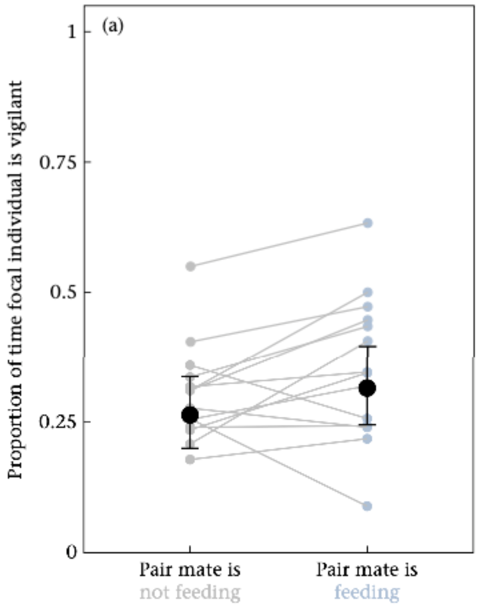

Importantly the analysis in this current study goes a step further. To analyze if marmosets can be modeled as coupled oscillators, we wanted to focus our analysis on the part of the data where this coupling is most likely. It is much more likely that animals can take into account the pair mate when they are close by, and the line of sight is not blocked by enrichment materials within the enclosure. The control condition of animals being alone was implemented to be able to calculate the amount of coupling that would be expected by chance. Even if animals are alone on the feeding basket, they have a certain frequency of changing from one behavioral state to the other (i.e., changing from vigilance to feeding). If the coupling that we get, when pairing the same pair mates together that live in the real groups *but* with the data from when they are alone on the feeding basket, we get a coupling constant that reflects just the chance level. In this data animals obviously *cannot* take each other into account; thus, we know just by having a certain

frequency of change (between the two states) of two distinct animals what would the "baseline" level of coupling amount to.
We now also include a new figure in the supplementary materials (see figure S5) to illustrate which data was used for which purpose.

- In the methods (lines 201 and following), the authors describe how they modelled vigilance in alone and together conditions. What about the other conditions? I would assume that also inside/outside should be relevant here.
Response: Yes, the methods under "Data preparation for analyses" apply to all four conditions. We have now clarified this in lines 508–510

> We used the onsets and offsets of behaviours "vigilance" and "feeding" coded from the videos (according to definitions in Table S2) to determine behavioural bouts of interest in all four conditions (inside 1 bowl, inside 2 bowls, outside 1 bowl, outside 2 bowls).

- Related to the previous point, the other conditions (1 vs. 2 bowls and inside vs. outside) are mostly reported in the supplementary materials. I suggest making this more clear and explicit in the main text.
Response: We find that very similarly to the already published study on the same data [68] that the conditions (1 vs 2 bowls, inside vs. outside) do have very little influence on the actual results of the modelling thus we refrained from putting the additionally illustrative figures about these results in the main text. Importantly all model results where "condition" was included are reported in the main text.

Minor:
-Line 45-46. The construction of the sentence especially after the colon is unclear. Please rephrase.
Response: We rephrased the sentence (lines 60-62):

> There is extensive evidence from invertebrates that synchronized patterns can emerge as epiphenomena of animals following very simple rules: fireflies, for example, simply flash sooner than usual whenever a neighbor flashes and the resulting synchrony emerges from these small changes in local interactions [19–21].

-Line 55-56: What do the authors mean by "non-human animals with more complex cognitive abilities"? Cognitive abilities were not explored up to that point in the manuscript, nor specific animal groups or species with "lesser" abilities. Please clarify.
Response: The paragraph from lines 59–71 is all about how animals with a less complex cognitive system compared to primates such as invertebrates can synchronize. We have added the word "cognitive" to line 59 to make this more obvious.

> However, the complexity of these phenomena does not necessarily imply complex cognitive mechanisms.

Then to the start of the next paragraph (line 73) we added specifically to what we compare more complex cognitive abilities:

> What remains underexplored is how flexible the synchronisation patterns are in non-human animals with more complex cognitive abilities compared to invertebrates.

We hope these small changes aid the reader to better understand what comparisons are made specifically in this part of the introduction.

-Lines 59-60: "are then confronted with novel regular and irregular sequences ("anisochrony detection") that they are expected to generalise the patterns of regularity too" – something is off with this sentence, please rephrase.
Response: We rephrased this section of the introduction to explain the reasoning behind these anisochrony detection tasks better, see lines 75–80:

> For example, typical rhythm perception tasks are performed as go/no-go tasks where animals are trained to respond to regular (isochronous) rhythms but not irregular (anisochronous) ones. The

same previously trained animals are then confronted with novel regular and importantly irregular sequences (“anisochrony detection”). Individuals are expected to generalise the patterns of regularity to the novel sequences, i.e., they should again respond to regular sequences but not to irregular ones.

-Line 62: please briefly explain what is meant by “approach behaviour”.
Response: We have added a short description of what approach behaviour means in these experiments, see line 82:

(i.e., moving closer to one of two sound sources)

-Lines 81-82 (“even without assumptions” …) is unclear. Please rephrase.
Response: We hope the rephrasing of the sentence is now clearer, see lines 100–102:

There is ample evidence that individuals living in bigger groups spend less time being vigilant [43–45], perhaps because they simply feel safer (i.e., the many eyes effect [46]) or because they actually take others’ vigilance into account.

-Line 96: “They changed their behavioural state more slowly when showing opposite behaviours” – can the authors clarify what this means?
Response: We have rephrased this sentence it now reads (see lines 115–117):

Furthermore, when feeding in proximity to their mate, they maintained the same behavior for longer periods of time when showing opposite behaviors, i.e., one individual being vigilant and the other feeding [68].

-Lines 132-133: the use of the past tense is inconsistent with the rest of the paragraph, please correct.
Response: Has been changed accordingly see line 187–189:

The extent of vocal accommodation is proportional to the initial differences in vocal properties, with higher initial acoustic differences between partners leading to more vocal accommodation later [79]

-Line 174: “Sessions were deemed finished when animals stopped eating because all the mash had been eaten, or when they interrupted feeding for more than 4 min. without resuming, or after 10 min.” – after 10 minutes from what? Please clarify.
Response: We clarified that the 10 min. are meant after the start of the session see lines 498-500:

Sessions were deemed finished when animals stopped eating because all the mash had been eaten, or when they interrupted feeding for more than 4 min. without resuming, or a maximum of 10 min after the start of the session.

-It would be helpful to have a picture or a figure describing the possible scenarios and the location of the experiment. This may also help in clarifying the “alone” control condition (see above).
Response: Thank you for this comment, we fully agree and now provide this figure in the supplementary materials (see figure S5). We refer to this figure both in the “Procedure” part of the Methods (see line 487)

For an overview of the experimental setup see figure S5.

as well as the “Data preparation for analyses” part of the methods (see line 510 onwards):

Analysis was restricted to behavioral states when (1) both animals were located on the feeding basket ('together'; to fit the Kuramoto Model and the non-periodic Kuramoto model, see below) or (2) one animal was located alone on the feeding basket ('alone'; for control simulations). Since animals were allowed to move freely in the enclosures, time spent on the basket occurred in bouts (see also supplementary figure S5).

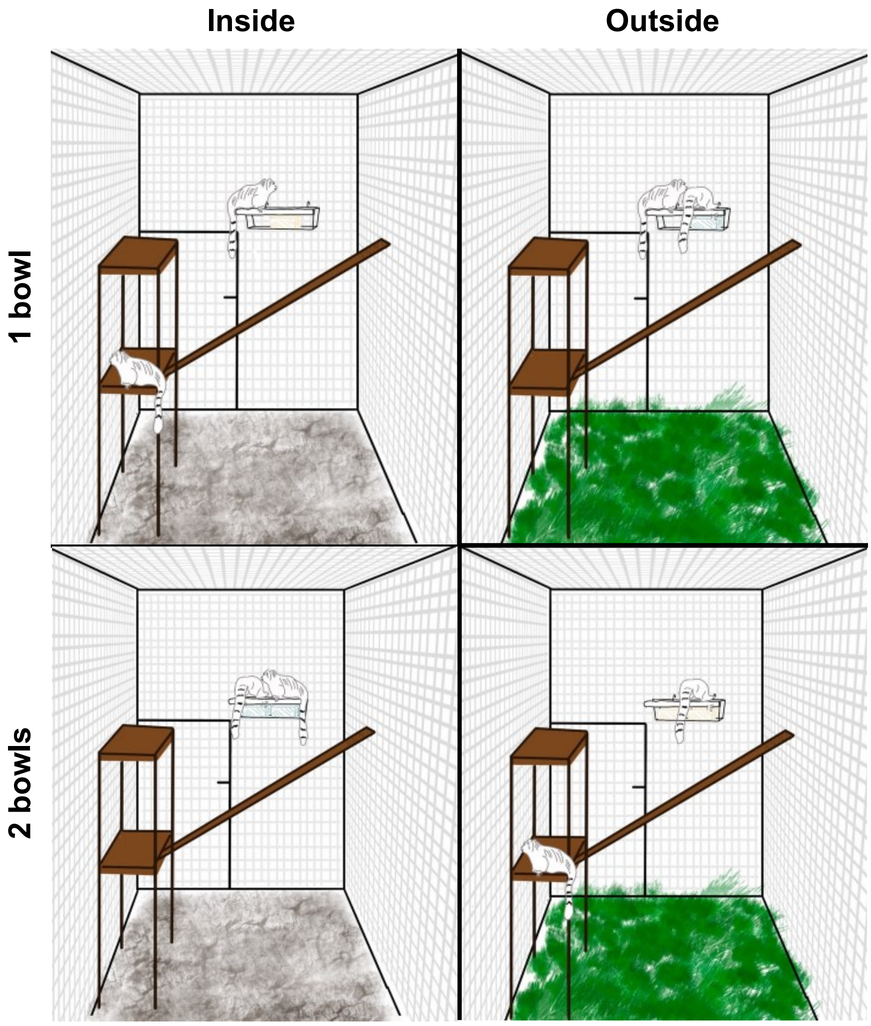

Figure S5 Overview of experimental conditions. This schematic shows the view from within home enclosures. Individuals experienced either of these conditions (inside 1 bowl, inside 2 bowl, outside 1 bowl, outside 2 bowls) in a randomized order (see table S1) in their respective home enclosures. Animal were fed from either 1 or 2 feeding bowls located inside a feeding basket on the front of enclosures. During the whole experimental duration, animals were able to move freely within the respective location. Cameras were place both outside and inside of home enclosures (not shown). *Video coding* was done frame by frame according to definitions from table S2 and specifically included all looking behavior over arms reach and not at a conspecific in any location of the enclosure as well as feeding behavior. For *data analysis* the datasets were restricted to either the "together" condition (blue bowls) where animals were situated together on the feeding basket or "alone" condition (yellow bowls) where animals were situated alone on the feeding basket. Note that even though "together" and "alone" conditions are only shown for two out of four possible conditions animals' data was present for all four conditions. Note that the marmosets are depicted disproportionally large relative to the size of the overall enclosures.

-Line 194: why was "out of sight" coded at all, if it was then mixed with the other transition behaviours?

Response: The coding for this study was done for the analysis published in the paper [68] where not only behaviours on the feeding basket are analysed (see also explanations above). On the feeding basket animals can hardly do many behaviours but this is different in other parts of the enclosure. The behaviour "out of sight" was thus carried over but in our opinion, it does not impair the analysis done in this current study.

-Line 371: please describe what was the null model.

Response: The null model was a model including the same response but the only predictor is the random intercept. We now include this specification (see lines 723-724):

Further, using ANOVA, we compared our model to the null model (only including the random intercept).

-Line 381: what exactly represents the variable "group"?
Response: Since we have multiple bouts per pair we controlled for these repeated and independent measurements at the level of the group with a random intercept. But since we only talk about pairs in the remaining manuscript we changed the phrasing to "pair id" to make it more clear (see line 720, as well as supplementary Table S3):

> Because the values followed a normal distribution (p<0.001, Kolmogorov-Smirnov test for normality), we fit a Gaussian linear mixed-effect model with location, number of bowls and the interaction between them as fixed effects and the pair id (according to Table S3) as the random intercept.

-Lines 484-486: "Overall, the fraction of bouts for which K-Lines 549 – 552: I am not sure I understand this passage. What does it mean that the marmoset remains in the current state for longer than usual? Please clarify.
Response: Marmoset pairs as coupled oscillators can only evolve to a state of anti-phase synchrony if their coupling constant K (a negative number) is less than Kc (a negative threshold). We have mentioned this while introducing K and Kc in our methods lines 590-592.

> A positive *K* indicates the tendency to attain synchrony in-phase and a negative *K* to attain anti-phase synchrony, given the magnitude of *K* is greater than a threshold - the critical coupling constant K_c.

The fact that K<Kc for more than 90% of actual bouts means that if given enough time, in all these instances, the marmosets would evolve into an anti-phase pattern of vigilance. We have now made this clear in line 361.

> It means in 92.1% of the cases, marmosets would evolve into anti-phase synchrony.

Reviewer #3:
Thank you for your submission on marmoset rhythms. Overall, your writing is clear and you have explained your use of the Kuramoto model well. I ask only for a couple of minor edits regarding Figure 5.
Response: We sincerely thank you for taking the time to go trough our manuscript and priving feedback. We have performed the minor edits according to your suggestions.

5A: On the vertical axis, there appears to be a middle state between V and F. Please clarify what this state is in the figure legend.
Response: The middle state is all the instances the animal's line of sight could not be classified by the experimenter as either vigilant or feeding (see out of sight in table S2). We have now made this clear in the figure legend.

> The state in between refers to all the instances when the animal's line of sight could not classified by the experimenter as either vigilant or feeding and was coded as 'out of sight' (see Table S2 for the definition).

5D: All three classes have a third column which corresponds to actual pairs in the non-periodic Kuramoto model fit. Please clarify why these column have weird shapes and what they represent. They look a bit strange when placed next to points and box plots.
Response: Figures 5C and 5D feature raincloud plots with original data points, boxplots summarizing the data and a half-violin plot showing the numerical distribution of the data. The third column is the half-violin plot. We have now explained this in the figure legend.

> **(D)** Raincloud plots depict the coupling constant (K) in control pairs, actual pairs with the classic Kuramoto model (CLK) fit and actual pairs with non-periodic Kuramoto model (NPK) fit. For each group, every point shown is a mean of K-values of all bouts of that pair (n=7 pairs), the box plots

summarize the statistics, and the half violin plots show the distribution of the data . ***p<0.001, post-hoc Nemenyi test. The bar plot on the top right shows the average mean absolute error (MAE) ± s.d. values in radians for CLK and NPK model fits. ***p<0.001, Wilcoxon signed-rank test.

We confirm that all authors have read and approved the changes made to the manuscript. We hope that the revised paper is suitable for inclusion in *PLOS Computational Biology* and we look forward to hearing from you.

On behalf of all co-authors,
Nikhil Phaniraj
